# Use of machine learning to classify adult ADHD and other conditions based on the Conners’ Adult ADHD Rating Scales

**DOI:** 10.1038/s41598-020-75868-y

**Published:** 2020-11-02

**Authors:** Hanna Christiansen, Mira-Lynn Chavanon, Oliver Hirsch, Martin H. Schmidt, Christian Meyer, Astrid Müller, Hans-Juergen Rumpf, Ilya Grigorev, Alexander Hoffmann

**Affiliations:** 1 Department of Clinical Child and Adolescent Psychology and Psychotherapy, Philipps University Marburg, Marburg, Germany; 2 FOM University of Applied Sciences, Birlenbacher Str. 17, 57078 Siegen, Germany; 3 Department of Social Medicine and Prevention, Institute for Community Medicine, University Medicine Greifswald, Greifswald, Germany; 4 Department of Psychosomatic Medicine and Psychotherapy, Hannover Medical School, Hannover, Germany; 5 Department of Psychiatry and Psychotherapy, University of Lübeck, Lübeck, Germany; 6 Statmath GmbH, Siegen, Germany

**Keywords:** Medical research, Outcomes research, Psychology, Human behaviour

## Abstract

A reliable diagnosis of adult Attention Deficit/Hyperactivity Disorder (ADHD) is challenging as many of the symptoms of ADHD resemble symptoms of other disorders. ADHD is associated with gambling disorder and obesity, showing overlaps of about 20% with each diagnosis. It is important for clinical practice to differentiate between conditions displaying similar symptoms via established diagnostic instruments. Applying the LightGBM algorithm in machine learning, we were able to differentiate subjects with ADHD, obesity, problematic gambling, and a control group using all 26 items of the Conners’ Adult ADHD Rating Scales (CAARS-S: S) with a global accuracy of .80; precision (positive predictive value) ranged between .78 (gambling) and .92 (obesity), recall (sensitivity) between .58 for obesity and .87 for ADHD. Models with the best 5 and best 10 items resulted in less satisfactory fits. The CAARS-S seems to be a promising instrument to be applied in clinical practice also for multiclassifying disorders displaying symptoms resembling ADHD.

## Introduction

Today there is general agreement that Attention Deficit/Hyperactivity Disorder (ADHD) often persists into adulthood with a prevalence rate of ~ 2.8% for adult ADHD^1,2^. According to the European Consensus Statement on diagnosing and treating adult ADHD^2^ the gold standard in diagnosis has four components: (a) a DSM-based clinical interview entailing the specific assessment of adult ADHD symptoms, (b) standardized questionnaires for assessing adult ADHD symptoms, (c) comorbidity assessment, and (d) the appraisal of school- or work certificates. This elaborate procedure might not be readily translatable in everyday clinical practice for various factors, e.g., economic restrictions, time limits regarding the diagnostic process or simply too little knowledge about adult ADHD^2^.

Another reason why adult ADHD is so hard to diagnose reliably is that many ADHD symptoms resemble those of other disorders^2^. Impaired concentration, a symptom shared with many disorders such as major depression, dysthymia, posttraumatic stress disorder or generalized anxiety disorder, might resemble the inattentiveness of adult ADHD. Restlessness and excessive talking, elements of ADHD’s hyperactivity component, may be difficult to differentiate from the psycho-motor agitation associated with mania, hypomania, major depressive disorder or generalized anxiety disorder. Impulsivity as apparent in ADHD may be difficult to distinguish from characteristics like those in manic or hypo-manic episodes, or from impulsive behavior inherent to borderline personality disorder and other disorders related to poor impulse control (e g., pathological buying, pathological gambling and compulsive sexual behavior).

In fact, adult ADHD is a highly comorbid disorder^2–5^. Specifically, disorders such as developmental disability, depressive disorder, and anxiety disorders^3^ as well as disorders associated with emotion dysregulation, a common symptom in ADHD^4,6^, co-occur with adult ADHD. For instance, personality disorders, especially borderline personality disorder (BPD) is closely associated with ADHD according to a recent review^7^. ADHD is also associated with pathological gambling, as about 20% of people with gambling disorder also experience ADHD^8^ and path analysis revealed emotion regulation to be a mediator between ADHD and gambling disorder^9^. To differentiate between pathological gambling and ADHD is important, as impulsivity due to ADHD might respond to psychostimulant treatment and thus attenuate the impairment associated with pathological gambling. A recent review on the connection between obesity and adult ADHD revealed that individuals with adult ADHD have higher odds of being overweight, a higher-than-average body mass index (BMI) score, and are significantly more often affected by obesity than subjects without ADHD; among patients seeking bariatric surgery, 20.9% had also been diagnosed with ADHD^10^. Again, reducing impulsivity through psychostimulant treatment could enhance obesity treatment. In summary, we highlight that the older the patient, the more difficult it might be to establish whether a patient with a history of inattention, hyperactivity, impulsivity, low self-esteem, and deficits in executive functions has ADHD, another disorder or both, since various other disorders might be associated with the deficits observed. Further, other disorders such as obesity and pathological gambling might be comorbid with ADHD and respond to psychostimulant treatment if diagnosed correctly.

There is a large body of literature on differentiating adult ADHD from healthy/community control groups—efforts that usually result in satisfying differentiation rates for rating scales^11–13^. However, problems arise when the aim is to distinguish adult patients with ADHD from other patient groups exhibiting similar symptoms, for instance anxiety. Under those circumstances, the specificity of rating scales is impaired when employed to inform a differential diagnosis^14^. With respect to BPD, common rating scales can differentiate patients with adult ADHD on the domain of severe impulsiveness, but not with respect to disturbed impulse control, disinhibition, hyperactivity or attentional control^15^. In general, false positive rates increase dramatically when using rating scale assessments. McCann and Roy-Byrne^12^ tested the ability of three different rating scales—the Adult Rating Scale (ARS), the Attention-Deficit Scale for Adults (ADSA), and the Symptom Inventory for ADHD (SI-ADHD)—to discriminate adult patients with ADHD from adult patients with major depression, bipolar disorder, anxiety disorders, and substance abuse/dependence disorders. Based on criterion cut-off scores of those inventories, individuals diagnosed with major depression or dysthymia yielded up to 73.9% false positives (up to 67.4% false positives across all other clinical patients). Solanto et al.^16^ tested the predictive value of the Brown Attention Deficit Disorder Scale and a Continuous Performance Test and concluded that sensitivity and specificity parameters provided no meaningful contribution to the differential diagnosis of ADHD and internalizing disorders.

The Conners’ Adult ADHD Rating Scales (CAARS) are well established and assess specific adult ADHD symptoms based on DSM-IV criteria with norms for males/females and four different age groups. The reported psychometric properties of the CAARS are highly satisfying, and they discriminate patients from healthy control subjects well (sensitivity 87%, specificity 85%, positive predictive value 85%, negative predictive value 87%, total correct classification rate 86%)^17^. Similar results have been found for translations of the CAARS, i. e. the German adaptation^13^. However, van Voorhees et al.^18^ examined the ability of the CAARS to differentiate between ADHD and other axis I disorders associated with attention problems, and found that patients with ADHD were likely to be indistinguishable from patients with anxiety and mood disorders when solely relying on the CAARS ratings-scales for diagnosis. Furthermore, there are gender effects demonstrating that females with ADHD are harder to differentiate from other patient groups than males^19^.

The goal of the current study was thus to establish whether the CAARS could discriminate between different patient groups presenting with symptoms similar to and/or frequently/accompanying adult ADHD, specifically disorders associated with emotion dysregulation and the lack of behavioral inhibition like obesity and problematic gambling. For control purposes, a healthy control group was added to the sample.

## Results

**Figure 1:**
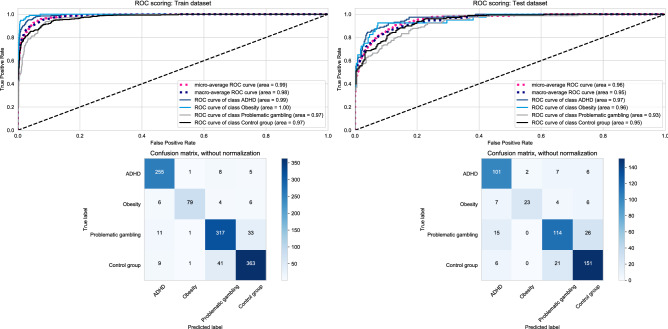
Classification-all features. Classification results for training and test data using all 26 items of the CAARS-S:S and age and gender.

Figure 1 displays our classification results for the training and test data using all 26 items of the CAARS-S:S and age and gender. Means and standard deviations of CAARS-S:S items and subscales of the different patient groups and the control group are displayed in Supplementary Table S1.

In the training data, individual AUC values were very high in all groups. The confusion matrix of the multiclassification problem in Fig. 1 resulted in a global accuracy of .89, a kappa coefficient of .84 which can both be regarded as very good agreement^20^ Precision (positive predictive value) ranged between .86 (problematic gambling) and .96 (obesity), recall (sensitivity) between .83 for obesity and .95 for ADHD, and the F1 score, which is the harmonic mean between precision and recall, ranged between .87 (problematic gambling) and .93 (ADHD). We can consider all these values high. Some misclassification occurred in that 33 (9.1%) of individuals with problematic gambling had been classified as controls and 41 (9.9%) of controls as individuals with problematic gambling.

In the test data, all groups revealed very high individual AUC values. The confusion matrix of the multiclassification problem in Fig. 1 resulted in a global accuracy of .80, a kappa coefficient of .71 which can both be considered good agreement^20^. Precision (positive predictive value) ranged between.78 (problematic gambling) and .92 (obesity), recall (sensitivity) between .58 for obesity and .87 for ADHD, and the F1 score, which is the harmonic mean between precision and recall, ranged between .71 (obesity) and .82 (controls & ADHD). These values can be regarded fair to high. Some misclassification occurred in that 26 (16.8%) of individuals with problematic gambling had been classified as controls and 21 (11.8%) of controls as individuals with problematic gambling.

**Figure 2:**
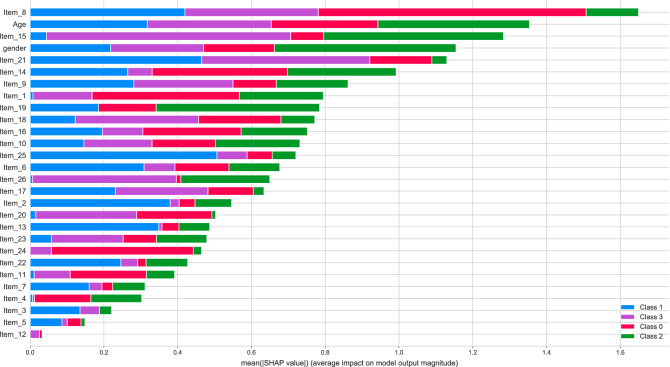
Feature importance-all features. Feature importance per group including all 26 items of the CAARS-S:S and age and gender. Class 0 = ADHD, Class 1 = obesity, Class 2 = pathological gambling, Class 3 = healthy controls.

The feature importance plot in Fig. 2 clarifies the differential importance of the variables we used for classification. In ADHD items 8 (angry outbursts), 1 (problems with interrupting people), and 24 (keeping focused on boring activities) were of special importance for classification. In obesity items 25 (lack of confidence), 21 (keeping focus), and 8 were most important. In problematic gambling items 15 (self-reproach), 19 (intruding in others’ activities), and gender were most important, and in controls items 15, 21, and 26 (learning experience) were considered most important.

We also applied logistic regression and support vector machines (SVM) to compare our results to other models. We report detailed results for our best fitting model. Among the three models, SVM and LightGBM demonstrate the best performance with slight fluctuations in metrics in favor of one or another, which partially depends on the split of the dataset (Table 1). It should be mentioned that accuracy alone is not an absolutely reliable metric. Therefore, some additional metrics like ROC curves should also be kept in mind. The confusion matrices are shown in Supplementary Tables S2-S4.

**Table 1:** Model comparison.

Model	Logistic regression		SVM		LightGBM	
Parameter	Train	Test	Train	Test	Train	Test
Micro-average ROC curve (area)	0.95	0.94	0.98	0.96	0.99	0.96
Macro-average ROC curve (area)	0.94	0.94	0.98	0.96	0.98	0.95
ROC curve of class ADHD	0.97	0.97	0.99	0.98	0.99	0.97
ROC curve of class obesity	0.96	0.94	0.99	0.96	1.00	0.96
ROC curve of class problematic gambling	0.91	0.91	0.97	0.94	0.97	0.93
ROC curve of class control group	0.92	0.92	0.97	0.95	0.97	0.95
Accuracy	0.8	0.79	0.89	0.82	0.89	0.80
Random seed (train/test split)	555		555		555	

Results of ROC curve analyses and accuracy of logistic regression, SVM, and LightGBM in our classification model using all 26 items of the CAARS-S:S and age and gender.

**Figure 3:**
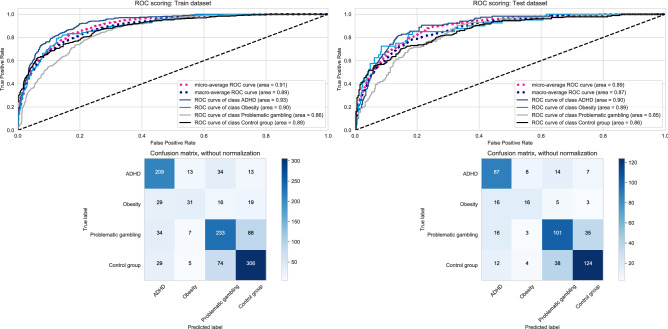
Classification-5 features. Classification results for training and test data using the best 5 features.

To optimize the model and eliminate redundant information, we calculated a model with just the best five overall features and reclassified the subjects in our sample. In the training data, individual AUC values were again very high in all groups. The confusion matrix of the multiclassification problem in Fig. 3 resulted in a global accuracy of .68, a kappa coefficient of .55 which can both be regarded as moderate^20^. Precision (positive predictive value) ranged between .55 (obesity) and .72 (controls), recall (sensitivity) between .32 for obesity and .77 for ADHD, and the F1 score, which is the harmonic mean between precision and recall, ranged between .41 (obesity) and .73 (ADHD & controls). These values can be considered low to moderate. A considerable amount of misclassification occurred in the obesity group. There was also misclassification in that 88 (24.3%) of individuals with problematic gambling were classified as controls, and 74 (17.9%) of controls as individuals with problematic gambling.

In the test data, individual AUC values were again very high in all groups. The confusion matrix of the multiclassification problem in Fig. 3 resulted in a global accuracy of .67, a kappa coefficient of .53 which can both be regarded as moderate^20^. Precision (positive predictive value) ranged between .52 (obesity) and .73 (controls), recall (sensitivity) between .40 for obesity and .75 for ADHD, and the F1 score, which is the harmonic mean between precision and recall, ranged between .45 (obesity) and .71 (controls). These values can be considered low to moderate. Some misclassification occurred in that 16 (40.0%) of subjects with obesity had been classified as having ADHD, 35 (22.6%) of individuals with problematic gambling were classified as controls, and 38 (21.3%) of controls as individuals with problematic gambling.

**Figure 4:**
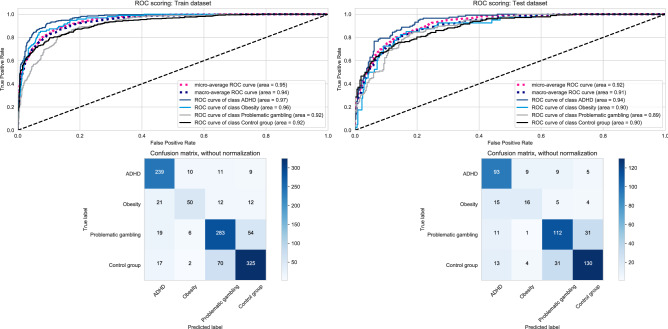
Classification-10 features. Classification results for training and test data using the best 10 features.

To optimize the aforementioned model, we calculated one with the best 10 overall features and reclassified the subjects in our sample. In the training data, all groups’ individual AUC values were again very high. The confusion matrix of the multiclassification problem in Fig. 4 resulted in a global accuracy of .79, a kappa coefficient of .70, which can both be regarded as good agreement^20^. Precision (positive predictive value) ranged between .74 (obesity) and .81 (ADHD & controls), recall (sensitivity) between .53 for obesity and .89 for ADHD, and the F1 score, which is the harmonic mean between precision and recall, ranged between .61 (obesity) and .85 (ADHD). These values can be regarded as fair to high. Misclassification occurred in the group with obesity, as 21 (22.1%) had been classified as individuals with ADHD. Misclassification also occurred in that 54 (14.9%) of individuals with problematic gambling had been classified as controls and 70 (16.9%) of controls as individuals with problematic gambling.

In the test data, individual AUC values were again very high in all groups. The confusion matrix of the multiclassification problem in Fig. 4 resulted in a global accuracy of.71, a kappa coefficient of.60 which can both be regarded as good agreement^20^. Precision (positive predictive value) ranged between.53 (obesity) and .77 (controls), recall (sensitivity) between .40 for obesity and .80 for ADHD, and the F1 score, which is the harmonic mean between precision and recall, ranged between .46 (obesity) and .75 (controls & ADHD). These values can be considered fair to good. Some misclassification occurred in that 15 (37.5%) of subjects with obesity had been classified as individuals with ADHD, 31 (20.0%) of those with problematic gambling had been classified as controls, and 31 (17.4%) of controls had been classified as individuals with problematic gambling.

A summary of model evaluation parameters for the three presented models can be found in Supplementary Table S5.

## Discussion

Our results demonstrate that with machine learning analyses, the various groups of patients we assumed to be similar in terms of impulse control and emotion regulation^4,9,10^ were very well distinguishable from each other as well as from the control group. The first data analysis using all 26 items of the CAARS:S and age and gender resulted in very high AUC values for all groups and revealed high accuracy of .80 as well as a high kappa coefficient of .71. These positive predictive values were fair to high in this analysis, as were the sensitivity values and harmonic mean F1. Correct classification rates were acceptable; 16.8% of individuals with problematic gambling had been falsely classified as belonging to the control group and 11.8% of the control group falsely classified as belonging to the group with problematic gambling. All further analyses revealed less satisfying classification rates, that is, attempts to optimize the model with just the best five and ten overall features were not as successful. With respect to the four different groups, various items in the CAARS-S: S proved important for classification. We found that for the control group, the items “failures in the past make it hard for me to believe in myself”, “being mentally absent in everyday activities” and “keeping focused on things” were most relevant for classification; the three items that emerged broadly for the group with ADHD target the core symptom domains of ADHD (temper-bouts, interruption of others, concentration problems); for people with obesity, temper-bouts and concentration were also most relevant, but further items concerning self-confidence (wish for more trust in one’s own abilities); for individuals with problematic gambling it was interference in other peoples´ activities and being highly self-critical were the most important classification items, as was male sex. The latter is probably due to the very high rate of males in this group (> 80%), one that exceeded those of the other three groups. In fact, the representative population-based study that provided the data for this study revealed that problematic gambling is a primarily male disorder, with significantly more males fulfilling more of this disorder’s criteria^21^. In childhood, significantly more boys (14%) than girls (6.3%) are affected by ADHD^22^; this difference remains significant in adult ADHD (OR 1.6), but the difference is not nearly as large (3.4% to 2.2%)^1^. With our study’s rate of 60.5% males with ADHD, this reflects the smaller difference in adulthood well, and contrary to former studies^19^ gender was not a relevant factor for classification. While other studies have demonstrated that differentiating females with ADHD in comparison to other patient groups with the CAARS was difficult^19^, we achieved such differentiation successfully in this study. The prevalence rates of obesity for men and women in Germany are similar^23^, though concerning morbid obesity (BMI ≥ 40 kg/m^2^), females’ rates (2.8%) are more than twice that of males (1.2%) in Germany according to a recent meta-analysis^24^, and our data. More females were enrolled in our control group, as is usually the case in voluntary studies^25^ like ours. Although the latter groups would have accommodated the expectation of sex differences, those only appear relevant when classifying problematic gambling.

Structured clinical interviews were applied to assess a broad range of disorders and were used in the different patient samples to establish primary disorders. As no specific information on ADHD symptoms is available apart from CAARS ratings, we performed additional descriptive analyses on groups with obesity, problematic gambling and healthy controls (Table S6, Supplementary Material) that show the percentage of individuals scoring 1.5 SD above the mean (T-distribution with a mean of 50 and a SD of 10) of the different CAARS subscales (inattention, hyperactivity, impulsivity, self-concept, ADHD Index). With respect to the ADHD Index as an overall marker of ADHD symptomatology, values range between 12% (males with obesity) to 24.3% (males with problematic gambling). This means that a substantial amount of individuals in the different groups score within the pathological range of the CAARS Index, and might indeed profit from treatment of comorbid ADHD symptoms. Specifically, even though such high scores are present in those groups, we were still able to successfully separate the groups with the machine learning approach applied.

An advantage of machine learning is that it is data driven and less sensitive to outliers^20,26^. Furthermore, it is a multivariate approach, as it does not rely on summary scores, but considers every single item. The risk of losing information is therefore minimized^26^. As can be seen in the model comparison table (Table 1), logistic regression performs slightly worse compared to SVM and LightGBM. At the same time SVM and LightGBM demonstrate really close results. Therefore, we had to choose between the two for the analyses. Using the LightGBM algorithm instead of SVM was based on the following considerations.

The performance of SVM depends heavily on the kernel used. If the solution is non-linear, the only correct choice is RBF kernel. There is also polynomial kernel but based on our own and other studies experience, polynomial kernels perform worse than RBF on average. Consequently, when conducting our analysis, we could not compare the performance of the RBF kernel with another kernel that is strong enough to really challenge it.

At the same time, LightGBM builds an ensemble of shallow and weak successive trees with each tree learning and improving on the previous. When combined, many weak successive trees produce a really powerful ensemble.

From our point of view, SVM's RBF kernel has too few parameters for tuning. In the case of real challenges that arise from the data, there is not enough leverage to adjust them. On the one hand, it means less time spent on tuning, but on the other hand, the goal of the paper was not to provide a fast solution but a precise and elaborated one. This is why we took our time and executed deep hyperparameter tuning for the bunch of the LightGBM’s hyperparameters.

In order to get correct and fair results from SVM, it is necessary to perform additional data transformation (like scaling, dummy variables generation etc.), which can be avoided by using LightGBM. Besides extra work this can make the quality of the forecast some kind of unstable and will highly depend on the values of the train dataset. To maintain good performance, SVM must be recalibrated and recalculated more often and much more attention must be paid to the value range of a newly incoming data set.

Based on our experience and results of many competitions, we would not use SVMs for really challenging real world problems. Anything beyond a lab problem might be better addressed with a different algorithm. We are not asserting that SVM is a poor algorithm, but the probability of it failing on a real-world problem is higher compared to some other more sophisticated algorithms.

Machine learning has been used in ADHD in order to differentiate subgroups with the help of neurophysiological measures. Based on power spectra EEG recordings during four different tasks, the support vector machine (SVM) method with tenfold cross-validation resulted in about 90% correct classifications between ADHD subtypes while the correct classifications between ADHD and controls was just about 70%. ADHD subgroups were regarded as being more homogeneous than the controls which should explain the differences^27^. In a visual GO/NOGO task, special ERP components were able to differentiate between adult ADHD subjects and controls with an accuracy of 92% using SVM with tenfold cross-validation^28^. Evaluating PET imaging and genetic predictors within the serotonergic system, an accuracy of .82 could be achieved for classification of ADHD and controls using Random Forest with fivefold cross-validation^29^. The Random Forest approach was used to investigate how multiple genetic and environmental factors jointly contribute to ADHD, or to examine whether hyperactivity persists in male and female adults with ADHD^30,31^. Teicher et al. were able to demonstrate that a computerized activity measure was best able to differentiate between adults with ADHD and controls (AUC = .83)^31^. This study’s promising results demonstrate that such analyses can be successfully applied to differentiating various patient groups with ADHD-like symptoms. Studies explicitly based on self-rating scales could not be found which stresses the importance for further research in this area. Connecting the literature to our analyses, we conclude that research groups in the area of ADHD relied predominantly on SVM, however the Random forest approach was also used^29^. It is known that in some challengeable cases GBMs may outperform Random Forest models. Based on the literature we therefore decided to go deeper and apply an even more sophisticated algorithm (LightGBM) trying to solve this issue.

To summarize our findings: the short version of the CAARS self-rating proved to be a useful instrument to classify different groups of patients that reveal overlapping symptoms in ADHD’s emotion-regulation and impulse-control domains^9^. Overall, only 13 patients with ADHD (5%) were misclassified in the first test data analysis using the complete feature set of the CAARS-S. This is an excellent rate and highly important, as the CAARS is known to have successfully differentiated patients from healthy controls^13^, but differential diagnoses proved much more difficult^18^, especially for females^19^ or other rating scales^12,16^. The classification with the best 5 and 10 features was not as successful, but that is not surprising due to the overlap between ADHD and obesity and between ADHD and gambling disorder that are both around 20% and the information based on only 5 or 10 features does then not prove to be sufficient. Nevertheless, the CAARS-S: self-rating seems to be a promising instrument to apply in clinical practise also for multiclassifying disorders displaying symptoms resembling those of ADHD.

### Limitations

We were unable to recruit patient groups to participate in this study apart from those with obesity and problematic gambling. As stated in our introduction, ADHD is highly comorbid^2^ and overlaps, for instance, with disorders in the internalizing (i.e., anxiety, depression)^3^ and externalizing spectrum (i.e., conduct disorder) as well as with personality disorders^4^. It would have been beneficial to have included such disorders. However, mental health disorders are prevalent both in patients seeking bariatric surgery (particularly depression, binge-eating disorder and anxiety disorder; meta-analysis prevalence estimates for current mood disorder 23%, binge-eating disorder 17%, anxiety disorder 12%)^32^ as well as in people with problematic gambling (with high comorbidity of axis I and axis II disorders). Of the individuals with problematic gambling in this study, the majority (> 80%) had another mental disorder according to axis I and about a third of this sample also on axis II^33^. As mentioned before, empirical data also reveal ADHD’s high prevalence among individuals with obesity^10^. Further, we only used a community convenience sample in whom we did not control for psychopathology symptoms and did not apply the DIVA-interview. We just asked whether a mental disorder had ever been diagnosed or not. Thus, a certain amount of noise is probable within this control sample. Our study results are relevant, as we were able to successfully differentiate our groups with great specificity despite our patients’ high comorbidity—thus highlighting the CAARS-S: S specificity.

Second, we only used the CAARS short self-rating version. Applying the long self- and observer CAARS version would also have been interesting, as would using different diagnostic instruments (e.g. structured clinical interviews, objective tests, measures assessing comorbid disorders) have been^2,12,16^. Future studies should thus aim to include groups of patients with a range of different disorders and diagnostic measures to test differential diagnostic properties.

## Materials and methods

### Sample

A total of n = 1629 subjects participated in the study and completed the short form of the CAARS (CAARS-S: S) consisting of 26 items. Of those, n = 1037 belonged to one of the clinical groups (ADHD: n = 385, obesity: n = 135, problematic gambling: n = 517), while n = 592 had no diagnosed disorder (referred to as our control group).

Patients with ADHD were individuals newly diagnosed at the adult ADHD outpatient clinic at Philipps University Marburg. They were all medication-naïve and examined by experienced, licensed clinical psychologists relying on a detailed clinical history, and the structured diagnostic interview for ADHD in adults (DIVA 2.0), a DSM-IV based clinical interview assessing the ADHD core symptoms in childhood and adulthood, as well as psychological domains often impaired in adult ADHD (https://www.divacenter.eu/Content/VertalingPDFs/German%20DIVA%202.0_FORM.pdf). The Conners’ Adult ADHD Rating Scales (CAARS-L self- and observer-ratings), and the Qb+^34,35^ were also used to confirm the diagnosis. The Amsterdam Short Term Memory Test (AKGT) was additionally applied as a symptom validity measure and to identify patients with severe attention problems^36^. The diagnosis was based on the DIVA 2.0 results in order to fulfill DSM-IV diagnostic criteria.

Data on people with obesity were gathered at the Department of Psychosomatic Medicine and Psychotherapy of the Hannover Medical School (MHH). They consisted of bariatric surgery candidates presenting morbid obesity (BMI ≥ 40 kg/m^2^) and were examined during their routine preoperative psychosomatic evaluation. This group’s mean BMI was 47.1 kg/m^2^ with a standard deviation of 8.47. Data regarding depressive symptoms were available for 123 of the 135 patients with obesity. Of those, 47.2% scored above the threshold for major depressive disorder of the Patient Health Questionnaire depression module. Overall, 41.5% of the sample met the criteria for binge eating disorder assessed with the German version of the Eating Disorder Examination interview. Gambling disorder as well as other potential comorbidities were assessed with the German version of the ICD module of the Structured Clinical Interview for DSM-IV research version (SCID-ICD). No gambling disorder was observed in any of the patients with obesity.

Subjects displaying problematic gambling behavior were recruited via a nationwide general population survey (n = 15,023) and from different populations with a high risk of gambling problems (gambling locations, via media announcements, outpatient addiction services, debt counselors, probation assistants, self-help groups and specialized inpatient treatment facilities)^21^. All participants were diagnosed according to the respective DSM-IV^37^ criteria by clinical professionals at the respective locations. Problematic gambling was diagnosed via the gambling section of the Composite International Diagnostic Interview^38^. Problematic gambling was defined by fulfilling at least one DSM-IV criterion for pathological gambling over lifetime. A total of 594 subjects from general or high‐risk populations participated in our in‐depth clinical interviews that also assessed other potential mental disorders, and 517 provided data sufficient to enable inclusion in our analysis. Among this sample with problematic gambling, n = 385 subjects fulfilled the DSM-IV criteria for pathological gambling.

All members of the community control group were gathered as a convenience sample at Philipps University Marburg; there was no thorough assessment, but participants were just asked whether a mental disorder was ever diagnosed or not. Table 2 illustrates details on our samples with the participants’ mean age and sex.

**Table 2:** Demographics.

Group	n	% of total	Male	Female	Age (SD)
ADHD	385	23.6	233 (60.5%)	152 (39.5%)	32.4 (9.9)
Obesity	135	8.3	41 (30.4%)	94 (69.6%)	39.9 (11.6)
Problematic gambling	517	31.8	416 (80.5%)	101 (19.5%)	41.2 (12.1)
Controls	592	36.3	227 (38.3%)	365 (61.7%)	34.2 (12.6)
TOTAL	1629	100.0	917 (56.3%)	712 (43.7%)	36.5 (12.3)

Demographic characteristics of the four subsamples.

### Measures

#### Conners’ Adult ADHD Rating Scales (CAARS-S: S)

The German adaptation of the CAARS (German version^39^: assesses ADHD core-symptoms and related problematic behavior in adults 18 years of age and older. Symptoms are rated on a Likert-scale from “0” (not at all/never) to “3” (very much/very frequently). The short forms of the instrument (CAARS-S/O: S) used in this study consist of 26 items assessing ADHD core-symptoms and self-concept (see Table 3 for the scale’s description). The short scale’s internal consistency is highly satisfactory (range: 0.77 (Hyperactivity/Impulsivity for males aged 18–29) to .90 (Self-Concept for males aged 50 and older) and re-test reliability were generally high (r_tt_ = .84–.91). We obtained factorial, concurrent and criterion validity for all scales^13^. Cronbach’s alpha values for this sample ranged from α = .76 to .81 and were satisfactory.

**Table 3:** CAARS-S:S.

No	Subscale	Item
	**Inattention/memory problems**	*α* = *.*76
1	Problems organizing oneself	3
2	Keeping track of several tasks	5
3	Finishing tasks	17
4	Procrastination	18
5	Keeping focus	21
	**Hyperactivity/restlessness**	*α* = *.*78
6	Problems with constantly moving	4
7	Getting bored easily	6
8	Sensation-seeking	10
9	Feelings of inner unrest	11
10	Fidgetiness	23
	**Impulsivity/emotional lability**	*α* = *.*81
11	Problems with interrupting people	1
12	Controlling temper	7
13	Angry outbursts	8
14	Irritability	13
15	Capriciousness	20
	**Problems with self-concept**	*α* = *.*79
16	Problems with self-efficacy	9
17	Self-reproach	15
18	Faking self-confidence	16
19	Lack of confidence	25
20	Learning experience	26
	**ADHD-index**	*α* = *.*77
21	Problems with restlessness	2
22	Distractibility	12
23	Being a low performer	14
24	Intruding in others’ activities	19
25	Hyper-focus	22
26	Keeping focused on boring activities	24

All Items (paraphrased) used in the CAARS short form.

### Statistical analyses

#### Machine learning

We applied machine learning to classify patients in the different samples and used all 26 items of the CAARS-S: S and age and gender for analyses. All calculations were performed in Python 3.6 using the LightGBM (Light Gradient Boosting Machine) algorithm and the hyperparameter tuning algorithm hyperopt. This is a gradient boosting framework that uses tree based learning algorithms. As LightGBM uses tree-based learning algorithm, the way of feature selection is identical to one of decision trees. In our model we used a Gradient Boosting Decision Tree algorithm that in turn typically used the classical CART (Classification and Regression Trees) algorithm that selects the split predictor, which maximizes the split-criterion gain over all possible splits of all predictors used on iteration. The exact parameters used are found in our Supplementary material (Table S7).

The data were split into training data (n = 1140, 70%) and test data (n = 489, 30%). We split our data on training and test datasets with a classical relationship 70/30 considering the stratification of our classes. The analysis was performed in such a way that neither during the training nor during the hyperparameter tuning the model has seen the test dataset, which served as “new” incoming data. The train dataset underwent an additional split under k-Fold cross validation during the hyperparameter tuning. K-Fold cross-validation by itself was not an object of a hyperparameter tuning, it was used in order to evaluate the generalization capacity of the selected hyperparameters. Number of folds was selected as 5 (default), which in this case corresponds to our empirical observation considering the desired generalization capacity and the sample size. Log loss for multi-class classification was used as an evaluation metric, 100 Monte Carlo simulations were undertaken. Individual Receiver Operating Characteristic Curves (ROC) were calculated per group and the Area under the Curve (AUC) was used to assess model performance^40^. To assess model performance of the multiclass problem, confusion matrices of the training and test data were calculated and parameters like accuracy, kappa coefficient, precision (positive predictive value), recall (sensitivity), and the F1 score as a harmonic mean between precision and recall were extracted^20^. Feature importance per group was examined and the most important five and ten features were used to build a classification model in the form of a further shortened test which was then evaluated as described above.

### Ethical statement

The authors assert that all procedures contributing to this work comply with the ethical standards of the relevant national and institutional committees on human experimentation and with the Helsinki Declaration of 1975, as revised in 2008. The study was approved by the local review board of the institute of psychology, Philipps University Marburg, as well as by the review boards of the departments of medicine at the Universities of Hannover (Hannover Medical School) and Greifswald. Written informed consent was obtained from all participants, and their confidentiality was assured. Subject data was collected from 2010 to 2013 through convenience sampling at the participating centers. All subjects were provided with a short study description and were asked to fill out the short CAARS self-rating questionnaire. For analyses, data were sent to the Department of Psychology at the Philipps University Marburg.

## Supplementary information

Supplementary Information

## Data Availability

The data is available upon request.
