# Supplementary material for: Use of machine learning to classify adult ADHD and other conditions based on the Conners’ Adult ADHD Rating Scales
Source: Sci Rep. 2020 Nov 2;10:18871. doi: 10.1038/s41598-020-75868-y (PMC7608669; doi:10.1038/s41598-020-75868-y)
Supplement: Supplementary file 1 — Supplementary Information [file 41598_2020_75868_MOESM1_ESM.docx]

Use of machine learning to classify adult ADHD and other conditions based on the Conners Adult ADHD Rating Scales

Short title: Classification of adult ADHD and other conditions

**Hanna Christiansen^1^, PhD, Mira-Lynn Chavanon^1^, PhD, Oliver Hirsch^2*^ , PhD, Martin H. Schmidt^1^, PhD, Christian Meyer^3^, PhD, Astrid Müller^4^, MD, Hans-Juergen Rumpf^5^, PhD, Ilya Grigorev^6^, MSc, Alexander Hoffmann^6^, PhD**

^1^Department of Clinical Child and Adolescent Psychology and Psychotherapy, Philipps University Marburg, Germany

^2^FOM University of Applied Sciences, Siegen, Germany

^3^Department of Social Medicine and Prevention, Institute for Community Medicine, University Medicine Greifswald

^4^Department of Psychosomatic Medicine and Psychotherapy, Hannover Medical School, Hannover, Germany

^5^University of Lübeck, Department of Psychiatry and Psychotherapy

^6^statmath GmbH, Siegen, Germany

**Supplementary Materials**

**Supplementary tables**

|  | group | | | | | | | |
| --- | --- | --- | --- | --- | --- | --- | --- | --- |
|  | ADHD | | obesity | | problematic gaming | | control | |
|  | Mean | SD | Mean | SD | Mean | SD | Mean | SD |
| Item 1 | 1.84 | .80 | 1.34 | .75 | 1.09 | .65 | 1.06 | .77 |
| Item 2 | 1.69 | .91 | .58 | .74 | 1.17 | .86 | 1.01 | .86 |
| Item 3 | 1.59 | .95 | 1.01 | .95 | 1.02 | .83 | .84 | .87 |
| Item 4 | 1.90 | .91 | .99 | .82 | .96 | .87 | .77 | .84 |
| Item 5 | 1.43 | 1.03 | .84 | .78 | .97 | .89 | .76 | .76 |
| Item 6 | 2.22 | .88 | 1.89 | 1.06 | 1.06 | .89 | 1.05 | .86 |
| Item 7 | 2.09 | .83 | .95 | .83 | 1.15 | .87 | .96 | .80 |
| Item 8 | 2.18 | .81 | 1.62 | 1.00 | .63 | .78 | .47 | .70 |
| Item 9 | 1.66 | 1.04 | 1.18 | .93 | .79 | .86 | .75 | .77 |
| Item 10 | 1.73 | 1.00 | .68 | .83 | 1.20 | .88 | .94 | .73 |
| Item 11 | 2.04 | .94 | .84 | .86 | 1.13 | .91 | .83 | .81 |
| Item 12 | 1.81 | .90 | 1.00 | .81 | 1.08 | .75 | 1.18 | .78 |
| Item 13 | 1.86 | .95 | .60 | .81 | 1.05 | .81 | .87 | .82 |
| Item 14 | 1.18 | 1.00 | .47 | .70 | 1.13 | .84 | 1.01 | .84 |
| Item 15 | 1.88 | .95 | 1.56 | 1.05 | .99 | .86 | 1.77 | .88 |
| Item 16 | 2.10 | .89 | .98 | .84 | 1.32 | .93 | 1.20 | .79 |
| Item 17 | 1.49 | .95 | .39 | .64 | 1.16 | .95 | 1.19 | .96 |
| Item 18 | 2.19 | .84 | 1.26 | .87 | 1.20 | .89 | 1.13 | .87 |
| Item 19 | 1.81 | .92 | 1.12 | .86 | .61 | .70 | .73 | .73 |
| Item 20 | 2.14 | .84 | 1.10 | .88 | .96 | .85 | .79 | .82 |
| Item 21 | .97 | .97 | .13 | .38 | .80 | .78 | .92 | .78 |
| Item 22 | 1.65 | 1.04 | .57 | .81 | 1.02 | .81 | .99 | .90 |
| Item 23 | 1.31 | .97 | .44 | .69 | .61 | .79 | .38 | .68 |
| Item 24 | 2.24 | .82 | .99 | .91 | 1.06 | .89 | .92 | .91 |
| Item 25 | 1.60 | 1.00 | .53 | .63 | 1.26 | .99 | 1.30 | .98 |
| Item 26 | 1.63 | 1.02 | 1.12 | .94 | 1.11 | 1.02 | .77 | .92 |
| Inattention | 7.68 | 2.86 | 3.64 | 2.03 | 5.15 | 3.14 | 4.84 | 3.27 |
| Hyperactivity | 9.20 | 2.68 | 4.84 | 2.20 | 4.96 | 3.09 | 3.97 | 2.81 |
| Impulsivity | 10.12 | 2.57 | 5.61 | 2.49 | 4.87 | 2.77 | 4.16 | 2.81 |
| Self concept | 8.88 | 2.70 | 5.36 | 2.49 | 5.46 | 3.82 | 5.78 | 3.41 |
| ADHD Index | 21.49 | 5.99 | 10.82 | 5.58 | 12.04 | 6.18 | 10.81 | 6.31 |

**Table S1.** Means and standard deviations of CAARS-S:S items and subscales of the different patient groups and the control group.

| Logistic Regression | | Parameter: solver = 'lbfgs', cv=5, scoring='neg_log_loss' | | | |
| --- | --- | --- | --- | --- | --- |
| **Test Dataset** | | | | | |
| **True** | ADHD | 96 | 2 | 12 | 6 |
|  | Obesity | 7 | 25 | 3 | 5 |
|  | Problematic gambling | 11 | 0 | 124 | 20 |
|  | Control group | 6 | 2 | 28 | 142 |
|  | | ADHD | Obesity | Problematic gambling | Control group |
|  |  | **Predicted** | | | |

**Table S2.** Confusion matrix of logistic regression using all 26 items of the CAARS-S:S and age and gender.

| SVM | | Parameter: C=7, decision_function_shape='ovr', gamma=0.01, kernel='rbf', probability=True. | | | |
| --- | --- | --- | --- | --- | --- |
| **Test Dataset** | | | | | |
| **True** | ADHD | 103 | 5 | 7 | 1 |
|  | Obesity | 7 | 26 | 3 | 4 |
|  | Problematic gambling | 7 | 0 | 123 | 25 |
|  | Control group | 5 | 0 | 26 | 147 |
|  | | ADHD | Obesity | Problematic gambling | Control group |
|  |  | **Predicted** | | | |

**Table S3.** Confusion matrix of SVM using all 26 items of the CAARS-S:S and age and gender.

| LightGBM | | Parameter: colsample_bytree~0.771, learning_rate~0.082, max_depth=3, min_child_samples=115, min_child_weight=5, num_leaves=28, reg_alpha=2, reg_lambda=0, subsample~0.216 | | | |
| --- | --- | --- | --- | --- | --- |
| **Test Dataset** | | | | | |
| **True** | ADHD | 101 | 2 | 7 | 6 |
|  | Obesity | 7 | 23 | 4 | 6 |
|  | Problematic gambling | 15 | 0 | 114 | 26 |
|  | Control group | 6 | 0 | 21 | 151 |
|  | | ADHD | Obesity | Problematic gambling | Control group |
|  |  | **Predicted** | | | |

**Table S4.** Confusion matrix of LightGBM using all 26 items of the CAARS-S:S and age and gender.

|  | Precision | Recall | F1 | Accuracy | Kappa |
| --- | --- | --- | --- | --- | --- |
| Model 1  26 Items | .78-.92 | .58-.87 | .71-.82 | .80 | .71 |
| Model 2  5 best features | .52-.73 | .40-.75 | .45-.71 | .67 | .53 |
| Model 3  10 best features | .53-.77 | .40-.80 | .46-.75 | .71 | .60 |

**Table S5.** Summary of model evaluation parameters.

| **Group** | **Inattention** | **Hyperactivity** | **Impulsivity** | **Self-concept** | **ADHD Index** |
| --- | --- | --- | --- | --- | --- |
| Obesity male | 9.8 | 19.5 | 24.4 | 14.6 | 12.2 |
| Obesity female | 13.8 | 19.1 | 25.5 | 13.8 | 19.1 |
| Pathological gambling male | 34.1 | 29.6 | 27.2 | 30.3 | 24.3 |
| Pathological gambling female | 27.7 | 29.7 | 16.8 | 24.8 | 20.8 |
| Control male | 26.0 | 19.4 | 15.9 | 25.6 | 18.1 |
| Control female | 26.0 | 16.4 | 14.0 | 23.8 | 18.9 |

**Table S6.** Percentages of T scores ≥65 in groups with obesity, pathological gambling and healthy controls for different CAARS subscales.

| Parameter | Value |
| --- | --- |
| colsample_bytree | 0.771 |
| learning_rate | 0.082 |
| max_depth | 3 |
| min_child_samples | 115 |
| min_child_weight | 5 |
| num_leaves | 28 |
| reg_alpha | 2 |
| reg_lambda | 0 |
| subsample | 0.216 |
| Monte Carlo simulations | 100 |
| nFold CV (Hyperparameter Tuning) | 5 |

**Table S7.** Hypertuning model parameters.

colsample_bytree – proportion of attributes for each iteration

learning rate- gradient boosting

max_depth – tree depth (3 levels)

min_child_samples – minimal number of samples in one leaf; 115 subjects were in a terminal node

min_child_weight – minimum number of samples that a node can represent in order to be split further

reg_alpha/reg_lambda – L1/L2 regularisation

subsample – proportion of samples in each iteration
